# Multi-stakeholder perspectives regarding preferred modalities for mental health intervention delivered in the orthopedic clinic: A qualitative analysis

**DOI:** 10.21203/rs.3.rs-2327095/v1

**Published:** 2023-01-30

**Authors:** Abby L Cheng, Ashwin J Leo, Ryan P Calfee, Christopher J Dy, Melissa A Armbrecht, Joanna Abraham

**Affiliations:** Washington University School of Medicine; Washington University School of Medicine; Washington University School of Medicine; Washington University School of Medicine; Washington University School of Medicine; Washington University School of Medicine

**Keywords:** Digital mental health intervention, Chronic musculoskeletal pain, Anxiety, Depression, Equity, Digital divide, Feasibility, Usability, Implementation

## Abstract

**Background:**

Although depressive and anxious symptoms negatively impact musculoskeletal health and orthopedic outcomes, a gap remains in identifying modalities through which mental health intervention can realistically be delivered during orthopedic care. The purpose of this study was to understand orthopedic stakeholders’ perspectives regarding the feasibility, acceptability, and usability of digital, printed, and in-person intervention modalities to address mental health as part of orthopedic care.

**Methods:**

This single-center, qualitative study was conducted within the orthopedic department of a tertiary care center. Semi-structured interviews were conducted between January and May 2022. Two stakeholder groups were interviewed using a purposive sampling approach until thematic saturation was reached. The first group included adult orthopedic patients who presented for management of ≥ 3 months of neck or back pain. The second group included early, mid, and late career orthopedic clinicians and support staff members. Stakeholders’ interview responses were analyzed using deductive and inductive coding approaches followed by thematic analysis. Patients also performed usability testing of one digital and one printed mental health intervention.

**Results:**

Patients included 30 adults out of 85 approached (mean (SD) age 59 (14) years, 21 (70%) women, 12 (40%) non-White). Clinical team stakeholders included 22 orthopedic clinicians and support staff members out of 25 approached (11 (50%) women, 6 (27%) non-White). Clinical team members perceived a digital mental health intervention to be feasible and scalable to implement, and many patients appreciated that the digital modality offered privacy, immediate access to resources, and the ability to engage during nonbusiness hours. However, stakeholders also expressed that a printed mental health resource is still necessary to meet the needs of patients who prefer and/or can only engage with tangible, rather than digital, mental health resources. Many clinical team members expressed skepticism regarding the current feasibility of scalably incorporating in-person mental health support into orthopedic care.

**Conclusions:**

Although digital intervention offers implementation-related advantages over printed and in-person mental health interventions, a subset of often underserved patients will not currently be reached using exclusively digital intervention. Future research should work to identify combinations of effective mental health interventions that provide equitable access for orthopedic patients.

**Trial registration:**

Not applicable.

## Background

Depressive and anxious symptoms negatively impact physical function and recovery after a wide variety of orthopedic procedures (1–7). Furthermore, there is a continuing shift in the United States to allow for innovative financial structures to facilitate clinicians, regardless of specialty, to address patients’ whole-person health (8, 9). As a result, orthopedic clinicians are increasingly motivated to offer mental health resources to their patients as part of a comprehensive musculoskeletal treatment plan (10–23). Mental health interventions can be delivered via a variety of modalities such as digital, printed, and/or in-person, and a knowledge gap remains in identifying which modalities are simultaneously: 1) feasible for orthopedic teams to deliver efficiently, 2) acceptable to patients and clinicians, and 3) scalable to deliver across diverse orthopedic practice models. Furthermore, it is essential to understand unique factors that influence whether orthopedic patients and clinical teams are willing to contribute to clinical trials to identify the most effective mental health interventions that are suitable to deliver in an orthopedic setting.

The primary purpose of this study was to understand orthopedic patients’ and clinical team members’ perspectives regarding the feasibility, acceptability, and usability of digital, printed, and in-person intervention modalities to address mental health as part of musculoskeletal care. A second purpose was to understand these stakeholders’ perspectives regarding the feasibility and acceptability of participating in mental health related research trials in the context of musculoskeletal care.

## Methods

This single-site qualitative study was approved by the Washington University IRB. Participants gave written or verbal consent, and they received a $40 stipend for participating. Participants were enrolled between January and May 2022, and data analysis was completed in September 2022.

### Participants

Participants from two stakeholder groups were recruited. The first group consisted of adult (18 years or older) patients who presented to a Washington University orthopedic specialist for treatment of ≥ 3 months of neck or back pain. This population was chosen because among patients who seek care for a musculoskeletal condition, people with chronic neck or back pain have a particularly high comorbid prevalence of depression and anxiety (24–27). Potential participants were identified by pre-screening orthopedic clinic schedules, and patients were purposively sampled to include: 1) adults across the age spectrum, 2) at least 50% of participants who self-identified as a woman and 25% who self-identified with a racial/ethnic minority group, and 3) patients who reported no, mild, and severe symptoms of depression and/or anxiety on the clinic’s standard care Patient-Reported Outcomes Measurement Information System (PROMIS) Computer Adaptive Test (CAT) Depression and Anxiety measures (28–30). The study was introduced to patients via a pre-visit phone call or in-person at their visit.

The second participant group consisted of Washington University orthopedic clinical team members including clinicians and support staff. Purposive sampling was used to ensure the group included: 1) clinicians from all adult orthopedic subspecialties, 2) early, mid, and late career physicians, 3) operative and non-operative specialists, 4) members of all clinical support roles present in the clinic (i.e., nurses and medical assistants), and 5) team members who self-identified as women and with racial/ethnic minority groups. The study was introduced to team members via e-mail.

### Interviews

After completing a demographic survey (31, 32), stakeholders participated in a one-on-one, approximately 30-minute interview. Interviews with patient stakeholders were conducted by a research coordinator with formal qualitative research training who has worked with orthopedic patients for 18 years (MAA). Interviews with clinical team members were conducted by a medical student with masters-level training in qualitative research (AJL). The lead researcher, who is a sports medicine physiatrist and manages chronic spine conditions (ALC), also participated in the initial interviews. All interviewers were overseen by a researcher with extensive qualitative methods experience (JA). Interviews were audio and video recorded and were conducted in person or via secure video conferencing technology, per the participant’s preference.

The interviews were informed by semi-structured interview guides that were drafted by the lead researcher (ALC) and then revised based on feedback from research team members including orthopedic surgeons and qualitative researchers (RPC, CJD, JA) (Additional file 1). The guides were pilot tested prior to the stakeholder interviews and were iteratively revised based on participant responses during the interviews.

All stakeholders were asked to describe their preferences and perceptions of feasibility, acceptability, and usability regarding modality options for mental health interventions that could/should be offered in the context of orthopedic care. They were specifically asked to at least comment on digital, printed, and in-person options. They also provided feedback regarding specific examples of one digital and one printed intervention. The digital intervention, called Wysa for Chronic Pain, is an evidence-based mental health app that addresses the interplay between mental health and chronic pain (12, 13, 33). It is a multi-component intervention that delivers cognitive behavioral therapy, mindfulness training, and sleep tools (e.g., meditations, sleep hygiene education) via a digital chatbot and real-time, text-based communication with human counselors. The printed intervention is a mental health resource guide developed by the research team. It was designed to maximize usability for older adults and people with limited literacy, and it was iteratively refined from stakeholder feedback provided during this study. The final guide is two double-sided pages and is titled, “Wellness Resource Guide.” The guide uses icons to assist users in quickly identifying resources which are in person, virtual/online, free, reduced cost, and/or a crisis hotline. Resources mirror the tools offered by Wysa for Chronic Pain, and some are intentionally inclusive and welcoming of people from diverse backgrounds. Each resource is accompanied by a brief description, physical and online contact information, and a QR code that links users to the resource’s primary online information site.

Patient stakeholders also completed usability testing for these digital and printed interventions. For the digital intervention, they were asked to complete onboarding and schedule a session with a counselor. For the printed intervention, they were asked to demonstrate how to engage with a resource on the guide which appealed to them. They provided qualitative feedback and rated each intervention on the System Usability Scale (SUS), which is scored 0-100 with higher scores being favorable and scores above 80.3 interpreted as receiving an “A” (34, 35). Finally, all stakeholders described factors which would influence whether they would contribute to a randomized controlled trial related to a mental health intervention introduced during musculoskeletal care.

### Data Analysis

A preliminary codebook was developed by the lead researcher (ALC) using a deductive coding approach based on the dimensions of feasibility, acceptability, and usability. Next, using inductive coding, the codebook was refined and finalized by two team members (ALC and MAA (patient interviews), or ALC and AJL (clinical team interviews)) after they reviewed a sample of interview transcripts. All transcripts were independently coded by those two team members. Participant recruitment continued from each stakeholder group until thematic saturation was reached. Coding was completed with NVivo 12 software (QSR International; Doncaster, Australia). Group discussion was used to resolve coding discrepancies and organize codes into final themes.

## Results

Of 85 patients approached, 30 (35%) participated (mean (SD) age 59 (14) years, 21 (70%) women, 12 (40%) non-White, median (range) pain duration 3.3 (0.5–40) years) (Table 1). Of 25 clinical team members approached, 22 (88%) participated (11 (50%) women, 6 (27%) non-White) (Table 2).

### Digital mental health intervention

Clinical team members perceived delivery of a digital mental health intervention to be feasible and appealing (Table 3). They especially expressed optimism that if patients experience improved mental health and ability to cope with pain, they may not rely as heavily on the clinical team to address these challenges, which many team members did not feel well-equipped to manage. However, team members and patients also voiced possible implementation challenges, including out-of-pocket costs for patients and the concern for medicolegal liabilities and non-orthopedic responsibilities out of the scope of practice for team members. The digital modality was largely acceptable to patients, but patient-reported interest varied based on their self-described tech-savviness and whether, at any given time, they felt a need for intervention and perceived benefit from using it. To be an acceptable intervention, orthopedic clinicians often expressed a need to first be presented rigorous evidence of effectiveness. For successful implementation, patients and team members also recommended: 1) providing patients with a printed “Getting started” informational handout, 2) offering a telephone support line to assist patients with app onboarding if needed (rather than relying on the clinical team for assistance), and 3) developing clear medicolegal policies and support paths which ensure orthopedic team members understand and work within their certified scope of practice. Patients scored the digital intervention with a median SUS score of 81.3, IQR 61.3–95.0, range 0-100 (n = 30). They demonstrated varied proficiency in navigating the intervention, and although not uniformly true, older patients frequently had more difficulty than younger patients with independently completing usability tasks. The most common usability barrier was that iOS (Apple iPhone) users often could not recall their App Store password, which interfered with their ability to download the app (even though the download was free).

### Printed mental health resource guide

Compared to a digital intervention, team members expressed relatively greater feasibility to incorporate delivery of a printed mental health intervention into their existing clinic flow (Table 4). Patients and team members also expressed strong enthusiasm for a printed intervention to better meet the needs of patients who are generally not “tech users” and of patients who particularly prefer tangible resources for mental health related matters. To ensure acceptability and successful implementation of a printed intervention, some patients and team members suggested that the intervention be offered in a variety of methods during the orthopedic encounter (e.g., in the waiting room, on patients’ online portals, directly from clinical teams, etc). However, many patients expressed they would be most likely to engage with a printed intervention if, as part of discharge instructions, a team member highlights the intervention components that are most recommended for them. Regarding usability, patients scored the printed resource guide similarly to the digital intervention, with a median SUS score of 87.5, IQR 65.6–92.5, range 45–100 (n = 30). Patients overwhelmingly perceived the final guide to be easy to use, but they also suggested: 1) creating an electronic version with active URLs to listed resources, and 2) de-emphasizing QR codes on the paper version of the guide so patients who are not familiar with QR codes do not feel overwhelmed.

### In-person mental health support

Although orthopedic patients and team members perceived that in-person mental health support would be the ideal intervention modality for some patients (e.g., with more severe psychological distress and/or a preference for in-person intervention), many team members expressed skepticism regarding the current financial and logistical feasibility of providing in-person support as part of musculoskeletal care (Table 5). If feasibility could be achieved, clinicians expressed various acceptable implementation options, such as: 1) incorporation of an in-person social worker into orthopedic clinics, or 2) preferred referral-based access to mental health clinicians who offer affordable, prompt appointment availability for patients referred from the orthopedic teams. The ideal method of incorporating in-person support was felt to vary depending on the patient population. Team members who more frequently manage chronic, life-changing (e.g., major traumatic or oncologic), and/or spine conditions expressed more interest in incorporating a mental health clinical team member into the orthopedic clinic (rather than relying on expedited referrals).

### Research considerations

Orthopedic patients and team members expressed overall feasibility and acceptability of conducting randomized controlled trials of mental health interventions delivered in the orthopedic clinic setting (Table 6). Although team members agreed that a brief introduction of the study by a clinical team member would increase patient recruitment for the study, clinicians expressed variable amounts of time (from none to essentially as much as needed) that they and their team members would be interested in and able to contribute. Patients generally expressed interest in participation in order to help other people and to access free, potentially helpful resources for themselves. Patients anticipated that barriers to sustained study participation could include: 1) episodes of reduced motivation and engagement with daily activities due to depressive and/or anxious symptoms, and 2) excessive study-related burden. Most patients expressed willingness to be randomized, although many patients also expressed a preference for one intervention over the other (e.g., digital or printed). Offering all study interventions to each patient by the end of the study increased patients’ enthusiasm for participating in a randomized trial.

## Discussion

In this study, we found that digital and printed modalities are both anticipated to be feasible and acceptable methods of delivering mental health intervention in the context of musculoskeletal care. Although implementation considerations slightly favored the printed modality, available evidence of intervention effectiveness currently favors the digital modality (36–38). Therefore, we anticipate that digital intervention can play a key role in facilitating delivery of mental health related therapeutic content to orthopedic patients, especially for patients who are younger and/or consider themselves to be proficient with and enjoy using mobile apps. At this time, also offering a printed intervention will likely be key to feasibly delivering content to a subset of often underserved patients, including many older patients, patients from rural communities with limited internet access, and those who cannot independently navigate mobile devices. Despite the feasibility challenges related to facilitating in-person mental health intervention, innovation and dissemination of successful models regarding care delivery to make this option possible will likely be most important for patients who are experiencing the most severe symptoms of depression, anxiety, and related impairment.

It is encouraging that orthopedic team members reported positive perceptions regarding the feasibility and acceptability of a digital intervention because: 1) this modality can provide at-home access to mental health tools when a patient’s mobility is limited due to a musculoskeletal condition, 2) there is growing evidence of effectiveness of digital mental health interventions, sometimes comparable to in-person mental health intervention (39, 40), and 3) there is increasing momentum for third party payers to subsidize digital interventions. Although some clinicians voiced medicolegal concerns related to offering a digital mental health intervention, the COVID-19 pandemic has accelerated the national push to facilitate seamless prescription of effective digital therapeutics, and we anticipate these concerns will lessen as clarity from governing bodies is achieved (41–44). As these system-level considerations are addressed, incorporating an evidence-based digital mental health intervention into orthopedic care has the potential to meaningfully contribute to the treatment plan for a substantial subset of orthopedic patients. However, a “digital divide” still exists, and offering only a digital intervention will not yet be an equitable solution. Many patients who are already at increased risk of poor outcomes, such as older adults and people from rural locations with less internet access, are those who are least likely to successfully engage with a digital mental health intervention (45, 46).

Although patients and team members expressed somewhat favorable feasibility and equity of a printed intervention compared to a digital intervention, there is currently weaker evidence regarding the clinical effectiveness of printed mental health interventions. So far, self-guided interventions have achieved small, yet still significant mean effects on mental health symptoms (meta-analysis d = 0.23, Number Needed to Treat (NNT) of 6.4) (38, 47). A subset of people have demonstrated high engagement with self-guided interventions, and low-intensity resource referral interventions have been shown to improve awareness and use of existing community resources (48). Given the feedback from our stakeholders, we hypothesize that offering a well-designed printed resource referral intervention to the subset of orthopedic patients who voice a preference for a printed rather than a digital intervention could: 1) improve the previously identified NNT, and 2) improve quality of life for this subset of patients, relative to what they would have achieved if they were offered an intervention with which they would not be able to engage at all (49).

Although our stakeholders also strongly favored the option of in-person, one-on-one mental health support for some patients, perceived financial and logistical barriers still substantially tamper enthusiasm for current feasibility. Orthopedic practices could circumvent clinic-facing financial barriers to in-person mental health support by developing a “preferred access” referral list to mental health clinicians in the community. However, due to restrictive third party payer policies and the nationwide shortage of mental health clinicians, patients would still face the same financial and wait-time barriers to accessing care that they currently face when independently seeking mental health support (50–52). One-on-one telehealth psychotherapy could be considered an alternative “in-person” support option. However, telehealth does not necessarily address the “digital divide” barrier, patient-facing financial barriers, or the widespread shortage of mental health clinicians. Telephone based support is another alternative modality that preserves the “human connection,” but this modality has not been well-received by subpopulations of orthopedic patients (16).

### Limitations

A limitation of this study is that all stakeholders were recruited from a single institution in a single metropolitan region. Therefore, some of the mental health resources listed on the printed intervention that we tested may not be available elsewhere, although many of the included resources are widely available virtually. Similarly, the feasibility of incorporating in-person mental health support will somewhat vary based on regional resources, although the shortage of mental health clinicians is a widespread problem nationally and globally (50–52). Also of note, all patients in this study presented for treatment of chronic neck or back pain. External validity of our patient-related findings needs to be assessed in other orthopedic patient populations who may have unique sociodemographic distributions and patterns of mobile device use (e.g., major orthopedic trauma, sports medicine, etc).

## Conclusions

In this study, we found that orthopedic patients and clinical team members perceive distinct advantages and challenges related to integrating digital, printed, and in-person modalities of mental health intervention into the orthopedic care setting. Digital intervention may currently have a favorable balance of feasibility and evidence of effectiveness compared to the other modalities, but an important, often underserved, subset of patients will not currently be reached using exclusively digital intervention. To reach as many patients as possible and to particularly engage patients who are older, from rural communities, and/or otherwise cannot meaningfully engage with a digital intervention, we propose that mental health intervention in the orthopedic setting cannot be a one-size-fits-all approach. Multiple intervention modalities are needed. The next step will be to build on this stakeholder feedback and conduct rigorous clinical trials to identify interventions that are feasible, acceptable, scalable, *and effective* at improving mental and physical health outcomes in orthopedic patients.

## Figures and Tables

**Table 1:** Characteristics of orthopedic patient stakeholders (N = 30).

Characteristic	N(%) or Median (range)
Age (years)	63 (30–78)
Gender	
Men	9 (30%)
Women	21 (70%)
Race	
Asian	2 (7%)
Black / African American	10 (33%)
White / Caucasian	18 (60%)
Ethnicity	
Hispanic	1 (3%)
Not Hispanic	29 (97%)
Healthcare coverage	
Private health insurance	9 (30%)
Medicare / Medicare advantage	15 (50%)
Medicaid / Medicaid replacement	7 (23%)
Smartphone use	
Independent to download and learn new apps	17 (57%)
Needs assistance downloading and learning new apps	6 (20%)
Smartphone user but not for apps	5 (17%)
Not a smartphone user	2 (7%)
Psychiatric history, reported in medical record	
Depression	9 (30%)
Anxiety	7 (23%)
PROMIS scores	
Depression	52.4 (34.2–69.5)
Anxiety	59.5 (38.3–73.3)

Abbreviation:

PROMIS (Patient-Reported Outcomes Measurement Information System).

**Table 2:** Characteristics of orthopedic clinicians and support staff stakeholders (N = 22).

Characteristic	N (%)
Clinical role	
Orthopedic surgeon	13 (59%)
Non-operative physician	3 (14%)
Nurse practitioner	2 (9%)
Nurse	2 (9%)
Medical assistant	2 (9%)
Physician rank	
Assistant professor	8 (36%)
Associate professor	5 (23%)
Professor	3 (14%)
Gender	
Men	11 (50%)
Women	11 (50%)
Race	
White / Caucasian	16 (73%)
Black / African American	1 (5%)
Asian	2 (9%)
Multi-racial	1 (5%)
Other	1 (5%)
Ethnicity	
Hispanic	1 (5%)
Not Hispanic	21 (95%)

**Table 3:** Themes regarding use of digital intervention to address mental health in the orthopedic care setting.

Theme	Representative quotes
Feasibility	
Appealing: The ease of referring a patient to an app is appealing to orthopedic clinicians and clinical support staff, especially if the added resource reduces how much the patient needs to navigate mental health and pain challenges through the orthopedic office. Ideally, the app could be somewhat customized to the orthopedic patient population, even to the relevant body part or surgery (e.g., post-operative precautions, activity progression).	“I think an app like this would be amazing for a huge portion of the patients that we have.” (Medical assistant) “I’m not saying to a patient, ‘I’m treating your depression with this.’ I’m telling the patient, ‘This is a resource that we have, that we use as an option to help improve the patient’s well-being.“’ (Physician) “This, to me, would not be very difficult to discuss and just provide the information. Like, ‘Here’s an app. This is a platform you can use, and we highly recommend it. By no means do you have to use it.’ I mean, it’s a conversation piece. It’s not like we’ve got to spend 30 minutes discussing this…And quite honestly, we spend a lot of time talking to patients about their pain and about how it’s affecting their lifestyle. And it may even take some of that off of us because now they’re using their app versus us.” (Nurse)
Persistent implementation concerns: Potential barriers to delivering a mental health app in the orthopedic care setting include: 1) out-of-pocket costs for patients, and 2) the concern for added medicolegal liabilities and responsibilities out of the scope of practice for orthopedic clinicians and team members.	“I’m on a fixed income, so any increase in my healthcare cost, I’ve got to monitor pretty closely. I’ve seen people on social security who budget – they don’t have an extra $5 to spare.” (Patient, 44-year-old White man) “[Maybe] you can get it from your health insurance, and they pay for it.” (Patient, 40-year-old White woman) “My first thought is that this would probably be a nice resource for patients. My second thought is, if you initiate some intervention or application, what kind of legal responsibility do you have based on that output? What I don’t want to do, personally, is increase my medicolegal risk on being responsible for intervening or providing outputs to patients where I have no knowledge base or expertise.” (Physician)
Acceptability	
Digital advantages: The app was appealing to orthopedic patients who: 1) were interested in self-help resources, 2) did not feel ready or interested in reaching out to a person for mental health assistance, and 3) wanted convenient access to on-demand resources.	“I think this is excellent because people have access and the ability to look it up and say, ‘Oh, I’m getting stressed out about this. What exercises do I do?’“ (Patient, 71-year-old Asian man) “I believe with the app, it’s a safety for those that choose not to get out to see someone face-to-face. Because even me going to counseling, I didn’t want the stigma of having to go to therapy… I had a family member that just said that she wouldn’t mind doing therapy if she can do it through text. And I was like, ‘How deep is that?’ Because a lot of things, sometimes people can’t verbalize or vocalize what it is they’re feeling, but they can write it down to you.” (Patient, 31-year-old Black woman) “Online resources are sometimes the only thing that patients have. I was in a wheelchair for months, and I just couldn’t go places. I was in so much pain. You go by what you can find online.” (Patient, 43-year-old White woman) “I love that they have times available that are really late. Because sometimes with my schedule, by the time I can actually sit down and focus on something, it’s 10:00 PM.” (Patient, 40-year-old White woman) “I think the app will be very helpful to have when it’s late or when it’s early morning and you’re not getting any sleep or something.” (Patient, 60-year-old White man) “For it to be here waiting for me, not having to try to navigate getting into a shrink and all of that nonsense with my primary – just any chance to introduce more mental healthcare, I think is good, honestly.” (Patient, 44-year-old White man)
Tech savviness dependent: Orthopedic patients, clinicians, and clinical support staff agreed that digital interventions such as smartphone apps are preferred by many patients. They tend to be more preferred by young and middle-aged adults and less appealing to patients who are not “tech savvy,” including many (but not all) older adults. Estimates for the proportion of clinicians’ patient populations who might be interested in a digital intervention ranged from 5–70% and clustered around 20–25%.	“[Patients] are on their phones a lot more. Everything’s going to their phones. Even when they’re in pain or if they’re miserable or something, their phones are a lot more accessible than a laptop or a piece of paper. I give them a whole packet and they’ll say, ‘I know you gave me some stuff and I wrote it down somewhere, but I don’t know where I put it.“’ (Medical assistant) “We have an online database for a particular surgery that we do, and I think 30% of my patients request paper surveys. Which is insanely high. For every other person in my division, it’s like 5–15%. So, it just tends to be my geographic location, I think, because it’s a lot of people from rural areas. They maybe don’t really like using their smartphone, so it’s a challenge. I think it’s going to be less of a challenge, and there are more and more elderly people that are used to these things, but that’s going to be your toughest population to hit with any kind of digital intervention – the elderly.” (Physician) “I would give it a shot because, like I say, we’re getting older, and we need to know how to mentally deal with our aches and pains. We really do.” (Patient, 70-year-old Black man) “I don’t know that I would use my phone that way.” (Patient, 67-year-old white woman)
Patient situation dependent: Patients expressed particular interest in a mental health app that addressed their coexisting orthopedic pain and limitations. Patients anticipated using a mental health app more frequently if they found it to be helpful, if they were having a flare of pain or depressed or anxious thoughts, and if the app’s interventions were short and succinct. Some, but not all, of patients also appreciated reminder notifications within the app, and some, but not all, desired their input into the app to be linked back to their medical record.	“I can think of so many people I know with chronic pain that would love this app, actually.” (Patient, 47-year-old Black woman) “Ultimately, it’ll be whether I continue to see results from it. But right now, I’m actually pretty excited. I’ve been waiting for something like this to link my mental health with the pain that I’m in. So yeah, I’m gung ho.” (Patient, 44-year-old White man) “Notifications, to be honest. And then also when I’m just experiencing pain, that’s when I think I would use it more.” (Patient, 64-year-old White woman)
Evidence dependent: Before recommending a mental health app to patients, orthopedic clinicians want details on the content and delivery of the actual intervention, and they want to be reassured of the quality of the intervention and how patients will perceive it. There is some concern regarding reliance on a chatbot to deliver an intervention.	“The big question that I would have is, ‘How does this compare to seeing a ‘real person’?” But this is presumably going to be better than nothing.” (Physician) “I feel like people still want to talk to people. I think having a licensed provider on the other end to chat with them is better than a bot.” (Physician)
Facilitators for implementation: Facilitators reported by orthopedic patients and clinicians for delivering a mental health app to orthopedic patients include: 1) a printed informational “Getting started” handout for patients, 2) centralized phone support to assist patients in onboarding to the app, and 3) clear liability policies and a support path which does not filter mental health related questions or crises to the orthopedic clinician.	“It’s not like [our staff] are going to go through it and help put the app on the patient’s phone and go through that. Anything extensive like that might be like, ‘Oh gosh, we don’t have time to set it all up and to actually get them going with it and that type of thing.’ So yeah, I think being able to have a printed ‘How-to’ thing – to give that to them would be, I think, helpful.” (Nurse practitioner) As an older person, I learn more through visuals. I’m finding that if I hear and see it, I can retain it better. If somebody talks to me and tells me how to get through the app, then that would be better for me. The verbal, as well as the instructional handout, would be great.” (Patient, 71-year-old Black woman) “I’m assuming there’s a back-end to this app with someone monitoring it…We’re treating the patient and they’re putting information out there that we’re not receiving or monitoring. And what happens if this app captures a problem?” (Physician)
Usability	
Varied proficiency: Although not universally true, some older and even middle-aged orthopedic patients expressed interest in using the app but had more difficulty than they anticipated navigating through the app. In contrast, some patients had no difficulty at all navigating to tools within the app, although these patients tended to be younger.	“It seemed pretty self-explanatory.…Nothing was confusing.” *When asked to schedule a session with the human coach*: “I wouldn’t know how to get to that… I’m not sure how I got here, but I guess I just keep going back.” (Patient, 78-year-old White woman) “It’s actually pretty clear, pretty cut and dry, which is good.” (Patient, 47-year-old Black woman)
Password recall: The most common barrier to patients using the app was that many iOS (Apple iPhone) users could not remember their App Store password and therefore could not immediately download the app, even though the download was free.	“I think they want me to enter…my Apple ID? I think I will have to go home and check it.” (Patient, 71-year-old Asian man)

**Table 4:** Themes regarding use of printed intervention to address mental health in the orthopedic care setting.

Theme	Representative quotes
Feasibility	
Superior to a digital intervention: Orthopedic clinicians and clinical support staff expressed that delivery of a printed intervention would be even quicker and easier to integrate into current workflows than delivery of a digital intervention.	“This would be, to me, like handing out a piece of paper on icing instructions. You can provide the resource for them to get more information, but this is more of a passive approach where patients, if they need it, can look at it. I think this would be more reasonable [than an app] because then I’m not providing their care. They’re able to go to this and say, ‘Hey, gosh, if I really want to do yoga training or whatever, I can go click on that.’ Or, ‘If I really truly need mental health resources and I don’t know how to get it, oh, that’s a nice resource.’ But it’s very passive. And I prefer that because then I don’t think any of that’s going to come back as me trying to provide care.” (Physician) “I think there are easier flows on this end [compared to an app]… My nurse could very easily print this out and hand this to the patient as she’s handing them all their other stuff for their appointment.” (Physician)
Acceptability	
Sometimes preferred over a digital intervention: Orthopedic patients, clinicians, and clinical support staff agree that a printed resource option is more appealing than a digital intervention for some patients. The printed intervention was especially appealing to orthopedic patients who: 1) are not frequent mobile device users, 2) prefer “tangible” information, and 3) prefer local, in-person support for mental health matters. Estimates for the proportion of clinicians’ patient populations who might be interested in a printed intervention was similar to estimates for the digital intervention.	“Someone like me that is not used to just looking at their smartphone or their iPad for everything – they might prefer [this guide] to the app.” (Patient 78-year-old White woman) “Some people just like paper.” (Patient, 46-year-old White woman) “I like this a lot, because again, it’s local. It’s resources within our city, and it’s easy. I like this. Honestly, I would prefer this to [an app], if it was a one-or-the-other…I like the idea of an app, and once I start using it, I might change my mind. But I like that this [guide] lists resources within the state I live. It’s like, tangible places that are conceivably here.” (Patient, 40-year-old White woman) “I think the same number as the app. I think I would give this to the same patients that I would try to set up with the app.” (Physician)
Engagement concerns: Potential patient-facing barriers to using a printed guide include: 1) affordability for the resources listed on the guide, and 2) the potential to lose the paper on which the guide is printed.	“For this [resource on the guide], you mention the fee is set on a sliding scale ranging $15 to $40. I think putting information about insurance and also the price has effect on our decisio…So one decision rule is, what is the price, not just, which kind of service [the resource] is offering.” (Patient, 30-year-old Asian man) “If there is a space in MyChart where people can find resources, or even send an e-mail out – because I lose paper.” (Patient, 35-year-old Black woman)
Facilitators for implementation: Orthopedic patients, clinicians, and clinical support staff suggested that a facilitator to delivering a printed intervention to patients could include making the handout available at multiple time points during the orthopedic encounter (e.g., in the waiting room, on the clinic’s public-facing website, as paperwork received at clinic discharge, and/or via patients’ online medical portal). It could be offered to all patients who screen positively for high symptoms of depression or anxiety, and/or it could be offered to patients who are identified by the clinical team to have symptoms of depression or anxiety that interfere with their orthopedic clinical care. Orthopedic patients largely prefer receiving the printed guide *after* their encounter with the clinician, as a response to their interaction with the clinical orthopedic team.	“You can leave something like this in the rooms and with flyers that they can post on the walls. And I mean, that’s something you put on the wall in the room that says, ‘Resources.’ If anybody wants to take a picture of it on their phones or go to the QR codes, they can.’ And that way they can also do it if they’re by themselves in the room.“ (Physician) “For our patients in our [more complex] clinics, we have a packet that we give to patients, so having this incorporated in that would be really helpful.“ (Physician) “I might not give it to the [straightforward] patient, but if they, on their own, are looking and find out that this is a resource for them – they may also have chronic pain [in another body part] that I’m unaware of or that wasn’t a focus of our visit – then they may avail themselves of this. So, I think having it for everybody, but not necessarily printing it out for everybody, is probably helpful.“ (Physician) “I also think that maybe you should put it on the MyChart app. (Patient, 35-year-old Black woman) “I’d want to see this after I see the doctor… And it could come from the doctor or the nurse.“ (Patient, 71-year-old Black woman)
Facilitator for use: Many orthopedic patients would prefer for the orthopedic clinical team to select and briefly discuss a few resources from the printed guide which they are most encouraged to pursue.	“I think it’s a good option, but I believe they have to explain some of the stuff, at least in the guidance. So it’s not like, just give [patients] the guidance and they read it later. Maybe give them some idea about how everything works, make some motivation for the people to use it. If [patients] have some extra information other than having just the guide, I think if you discuss it, maybe they take it more seriously and do one of the steps.” (Patient, 30-year-old Asian man) “I think the provider would need to probably circle one or two things they want the person to do because I think if you just hand them this resource list, I feel like they’re not going to do anything, or they’re not really going to know what to do. I think if you have one thing you want them to do and point them to that, there’s a higher chance they’ll actually do it. But I think this is a great resource for providers, too – like a menu box of which ones we’re going to choose for this particular patient, something like that.” (Physician)
Usability	
Format preferences: Orthopedic patients, clinicians, and clinical support staff preferred that a printed guide be no longer than two double-sided pages, with large font, simple language, bullet points, clear cost information, bold colors, and an intuitive, yet pleasing format. When delivered electronically, URLs should be active hyperlinks. When delivered on paper, QR codes can be included on the guide to facilitate access to resource URLs, but QR codes may overwhelm and deter some patients from further exploring the guide.	“I like that it’s narrowed down. Because I’ve looked for stuff like this before, and if you Google it, it’s overwhelming. Because you have so many options and it’s like, ‘How do I boil it down?“’ (Patient, 40-year-old White woman) “This is paper. If this were electronic and these were clickable, I might find it more useful. I could click on [a resource], and I wouldn’t have to type it in.” (Patient, 70-year-old Hispanic woman) “I think this would be great. I think the only drawback I see with this would be our patients that are not tech savvy. I just learned how to use QR codes. I’m 40, so I don’t think… I don’t know that my 75-year-old mom could use the QR code. I don’t know. So I think that just making sure it’s all-around age friendly. Making sure it’s functional and easy for those patients who may not be tech savvy is going to be a big priority.” (Medical assistant)

**Table 5:** Themes regarding incorporation of in-person support to address mental health in the orthopedic care setting.

Theme	Representative quotes
Feasibility	
Skepticism: Many orthopedic clinicians questioned the financial and logistical feasibility of in-person support from a mental health specialist within the orthopedic clinical environment.	“I think [a counselor or social worker] would be very helpful, but I don’t see it happening in this day and age in healthcare.” (Physician) “I don’t think there are enough patients, at least in my practice, to make it work out.” (Physician)
Acceptability	
Ideal for some patients: Orthopedic clinicians, clinical support staff, and patients expressed that a subset of patients require and prefer one-on-one in-person mental health support.	“I think in-person options are going to be the key. Handouts are great, but then they’re like, ‘Okay, now what? Are you going to schedule me with somebody? Is there somebody I can talk to? If you can’t help me, who’s going to help me? My primary care provider doesn’t want to deal with this. What do I do now? This isn’t a pain management doctor problem. So now what do I do?’“ (Medical assistant) “For a lot of patients, I’ve told them that they need to see or try to find a psychiatrist, but they always have trouble finding one.” (Physician) “I think it would be awesome to have a therapist that will come in and speak to you for maybe five, ten minutes that can give pointers, things that you can do to help, say, if a person needs it.” (Patient, 35-year-old Black woman) “I like to see them face to face. I like to have the interaction where you can see my face. You could follow up with a phone call or a computer, but initially I would like to have a face, a Zoom call, or something so you could see the expression on my face.” (Patient, 71-year-old Black woman)
Considerations for various care models: Orthopedic clinicians proposed various models to integrate in-person mental health support into the orthopedic care plan. Compared to orthopedic teams that care for relatively acute and correctable conditions, clinicians and support staff who predominantly care for patients with chronic conditions, spine conditions, and/or life-altering (e.g., major traumatic or oncologic) conditions more frequently expressed that a departmental social worker or counselor would be an important resource, as opposed to referral to an outside resource.	“There are a couple applications for social workers that I think would be helpful. One would be mental health counseling. The other is for patients that are uninsured or underinsured to give them resources and help with things. So, I think there’s utility for multiple roles that someone like a social worker can play, and I think that would be very beneficial. It can be one [social worker] at each clinical location. We’re not asking them to take on all of our patients. It’s a subset for sure.” (Physician) “Just a list of names of people that maybe we have a relationship with – a psychiatrist that maybe we can refer them to would be nice. Maybe forming some relationships with some psychiatrists. I think that, and then maybe some psychologists, as well.” (Physician) “I think it depends on the clinic. I mean, maybe in some ways you could consider having a ‘complex patient’ clinic and have more resources available there. Have longer appointment times, more resources available – like a psychologist or psychiatrist – at those visits.” (Physician)

**Table 6:** Themes regarding considerations for conducting mental health related research in the orthopedic care setting.

Theme	Representative quotes
Feasibility	
Clinical team interest and facilitators for recruitment success: Most orthopedic clinicians would be interested in contributing to a mental health related trial if participation is convenient such that: 1) the eligibility criteria are well-defined, 2) a dedicated research member is present real-time to complete the vast majority of the recruitment and enrollment activities, 3) frequent reminders are sent to clinicians as needed, 4) the study does not interfere with other ongoing studies, and 5) enrollment does not slow down clinic flow (e.g., due to space limitations). Study advertisements in the patient rooms would help patients initiate the conversation and would remind clinicians to discuss it, as well.	“The less work that we have to do in clinic to enroll somebody and the less it slows us down, the more likely I’m going to be to enroll patients. Then also make it very clear and easy to identify inclusion and exclusion criteria, because that’s always a difficult thing to remember in a busy clinic.” (Physician) “I think there has to be somebody to do it, whoever that is. There has to be a person for whom it’s on their radar. I feel like most of the time, my brain is full or empty or whatever. It usually can’t handle much more, you know?” (Physician) “If my team knows, I can ask them ahead of time to identify the patients. They can put a note in the appointment comment, or they can remind me as I’m running down the hallway to my next room. And then have something in the rooms, like a little a flyer in a brochure holder. Because then I have a visual reminder in the room when I’m engaging with the patient.” (Nurse practitioner) “I think it’s important to know that a lot of patients at [this institution] are enrolled in a lot of different studies. There’s a lot of concern when you introduce a new study that it’s going to impact the results of another study, which may be industry funded.” (Physician)
Patient interest and facilitators for enrollment success: Orthopedic patients report that motivators to participate in a mental health related randomized controlled trial in an orthopedic care setting include: 1) a desire to improve their pain and function, and 2) a desire to help people. Many orthopedic clinicians believe patients would be more likely to participate in the research study if: 1) the clinician voices support for the study and recommends it, 2) the topic is introduced in an approachable and compelling manner, and 3) the added burden is minimal.	“For me, the interesting part of this is I feel like I’m suffering from mental issues, and at the same time, have the pain. So if there is really a relationship between these two and if there is a solution, I really would like to know. That’s the reason to participate for me.” (Patient, 30-year-old Black woman) “I think it’s important for somebody on the [clinical] team to mention the study to the patient. ‘Hey, we’re doing this study. We think it can be potentially really helpful. Would you mind talking to the research coordinator?’“ (Physician) “I think just the presentation – having them feel accepted and that this is really looking at an overall perspective for health and wellness…that we’re on their side and they’re not feeling, I guess, judged or bad. Taking a more positive approach to it. I think that delivery would be helpful for them to accept it.” (Nurse practitioner) “If it were a lot of time or a lot of effort for anyone, they would not be okay with it.” (Physician)
Patient barriers to participation: Patients reported the following anticipated barriers to participation: 1) worsening of mental health such that the patient loses motivation and energy to participate in typical daily activities, or 2) excessive burden from the study.	“One, if I can afford it, and two, if I can get there. And three, if my mental capacity is willing to take this on that day. But yeah, I definitely would [participate].” (Patient, 35-year-old Black woman) “If you catch me while I’m down and not feeling great about things, it would be harder to find the motivation to try something new, even if it would be helpful.” (Patient, 44-year-old White man)
Acceptability	
Acceptability of randomization: Generally, orthopedic clinicians believe patients would be willing to participate and be randomized to one of a variety of mental health interventions. While most patients expressed willingness to be randomized, many also expressed a preference for one intervention over the other (e.g., digital or printed). Offering all interventions by the end of the study period increased the appeal to many patients.	“I think patients are less likely to participate and be randomized into interventions where they have a vested interest and a clear prejudice for a certain outcome… Trying to get somebody to commit to a randomization for surgery is very difficult because they may have strong feelings, but for this, I don’t think that they’re going to feel strongly about it.” (Physician) “To me, either one would be okay.” (Patient, 71-year-old Black woman)

## Data Availability

The qualitative datasets generated and analyzed in the current study are not publicly available to protect the identities of participants. Data may be available from the corresponding author on reasonable request.

## References

[1] Zalikha AK, Karabon R Hajj Hussein I, El-Othmani MM. Anxiety and Depression Impact on Inhospital Complications and Outcomes After Total Knee and Hip Arthroplasty: A Propensity Score-Weighted Retrospective Analysis. J Am Acad Orthop Surg. 2021;29(20):873–84. 34525481 10.5435/JAAOS-D-20-00721

[2] Henry JK, Barth K, Cororaton A, Hummel A, Cody EA, Mancuso CA, Association of Depression and Anxiety With Expectations and Satisfaction in Foot and Ankle Surgery. J Am Acad Orthop Surg. 2021;29(16) :714–22. 34142981 10.5435/JAAOS-D-20-01394

[3] Cronin KJ, Mair SD, Hawk GS, Thompson KL, Hettrich CM, Jacobs CA. Increased Health Care Costs and Opioid Use in Patients with Anxiety and Depression Undergoing Rotator Cuff Repair. Arthroscopy. 2020;36(10):2655–60. 32497659 10.1016/j.arthro.2020.05.038 PMC7554073

[4] Celestin J, Edwards RR, Jamison RN. Pretreatment psychosocial variables as predictors of outcomes following lumbar surgery and spinal cord stimulation: a systematic review and literature synthesis. Pain Med. 2009;10(4):639–53. 19638142 10.1111/j.1526-4637.2009.00632.x

[5] Knapp R Layson JT, Mohammad W, Pizzimenti N, Markel DC. The Effects of Depression and Anxiety on 90-day Readmission Rates After Total Hip and Knee Arthroplasty. Arthroplast Today. 2021 ;10:175–9. 34458530 10.1016/j.artd.2021.06.013 PMC8379358

[6] Cheng AL, Schwabe M, Doering MM, Colditz GA, Prather H. The Effect of Psychological Impairment on Outcomes in Patients With Prearthritic Hip Disorders: A Systematic Review and Meta-analysis. The American journal of sports medicine. 2019:363546519883246-. 10.1177/0363546519883246 31829034

[7] Miner H, Rijk L, Thomas J, Ring D, Reichel LM, Fatehi A. Mental-Health Phenotypes and Patient-Reported Outcomes in Upper-Extremity Illness. J Bone Joint Surg Am. 2021 ;103(15):1411–6. 34357891 10.2106/JBJS.20.01945

[8] Centers for Medicare & Medicaid Services. Public Comment Summary Report: Clinician-Level and Clinician Group-Level Total Hip Arthroplasty (THA) and/or Total Knee Arthroplasty (TKA) Patient-Reporuted Outcome-Based Performance Measure (PRO-PM) 2022. Available from: https://mmshub.cms.gov/sites/default/files/PC-Summary-THATKA-PROPM.pdf. Accessed 1 October 2022.

[9] Siddiqi A, White PB, Mistry JB, Gwam CU, Nace J, Mont MA, Effect of Bundled Payments and Health Care Reform as Alternative Payment Models in Total Joint Arthroplasty: A Clinical Review. J Arthroplasty. 2017. 10.1016/j.arth.2017.03.027 28438453

[10] Ring D, Vranceanu AM. Editorial Comment: Papers from the International Consortium for Mental and Social Health in Musculoskeletal Care. Clin Orthop Relat Res. 2022;480(2):246–7. 34919062 10.1097/CORR.0000000000002098 PMC8747603

[11] Vranceanu AM, Bakhshaie J, Reichman M, Ring D. A Call for Interdisciplinary Collaboration to Promote Musculoskeletal Health: The Creation of the International Musculoskeletal Mental and Social Health Consortium (I-MESH). J Clin Psychol Med Settings. 2021. 10.1007/s10880-021-09827-8 PMC11863265 34605997

[12] Leo AJ, Schuelke MJ, Hunt DM, Metzler JR Miller JR Areán PA, A Digital Mental Health Intervention in an Orthopedic Setting for Patients With Symptoms of Depression and/or Anxiety: Feasibility Prospective Cohort Study. JMIR formative research. 2022;6(2):e34889. 35039278 10.2196/34889 PMC8902664

[13] Leo AJ, Schuelke MJ, Hunt DM, Miller JR Areán PA, Cheng AL. Digital Mental Health Intervention Plus Usual Care Compared With Usual Care Only and Usual Care Plus In-Person Psychological Counseling for Orthopedic Patients With Symptoms of Depression or Anxiety: Cohort Study. JMIR formative research. 2022;6(5):e36203. 35507387 10.2196/36203 PMC9118017

[14] Jacobs CA, Mace RA, Greenberg J, Popok PJ, Reichman M, Lattermann C, Development of a mind body program for obese knee osteoarthritis patients with comorbid depression. Contemp Clin Trials Commun. 2021 ;21:100720. 33553798 10.1016/j.conctc.2021.100720 PMC7859301

[15] Bakhshaie J, Doorley J, Reichman M, Mace R, Laverty D, Matuszewski PE, Optimizing the implementation of a multisite feasibility trial of a mind-body program in acute orthopedic trauma. Transl Behav Med. 2022;12(5):642–53. 35195266 10.1093/tbm/ibac004 PMC9154268

[16] Vranceanu AM, Jacobs C, Lin A, Greenberg J, Funes CJ, Harris MB, Results of a feasibility randomized controlled trial (RCT) of the Toolkit for Optimal Recovery (TOR): a live video program to prevent chronic pain in at-risk adults with orthopedic injuries. Pilot Feasibility Stud. 2019;5:30. 30820341 10.1186/s40814-019-0416-7 PMC6381627

[17] Ring D. Mental and Social Health Are Inseparable from Physical Health. J Bone Joint Surg Am. 2021 ;103(11 ):951–2. 33877056 10.2106/JBJS.21.00121

[18] Ring D. Priorities for Advancing Mental and Social Health Among People Presenting for Care of Musculoskeletal Symptoms : International Consortium for Mental and Social Health in Musculoskeletal Care. J Clin Psychol Med Settings. 2022. 10.1007/s10880-022-09865-w 35318572

[19] Vranceanu AM, Bakhshaie J, Reichman M, Doorley J, Mace RA, Jacobs C, Understanding barriers and facilitators to implementation of psychosocial care within orthopedic trauma centers: a qualitative study with multidisciplinary stakeholders from geographically diverse settings. Implementation science communications. 2021;2(1):102. 34526133 10.1186/s43058-021-00208-8 PMC8441236

[20] Vranceanu AM, Beks RB, Guitton TG, Janssen SJ, Ring D. How do Orthopaedic Surgeons Address Psychological Aspects of Illness? The archives of bone and joint surgery. 2017;5(1):2–9. 28271080 PMC5339350

[21] Reichman M, Bakhshaie J, Grunberg VA, Doorley JD, Vranceanu AM. What Are Orthopaedic Healthcare Professionals’ Attitudes Toward Addressing Patient Psychosocial Factors? A Mixed-Methods Investigation. Clin Orthop Relat Res. 2022;480(2):248–62. 34779793 10.1097/CORR.0000000000002043 PMC8747600

[22] Sagar I, Popok PJ, Reichman M, Lester EG, Doorley J, Bakhshaie J, Orthopedic Providers’ Preferences for Education and Training on Psychosocial Clinical Research Initiatives: A Qualitative Investigation. J Patient Exp. 2022;9:23743735221092570. 35450087 10.1177/23743735221092570 PMC9016593

[23] Cheng AL LA, Calfee RR Dy CJ, Armbrecht MA, Abraham J. What Are Orthopaedic Patients’ and Clinical Team Members’ Perspectives Regarding Whether and How to Address Mental Health in the Orthopaedic Care Setting? A Qualitative Investigation of Patients With Neck or Back Pain. Clin Orthop Relat Res. 2022;In press. 10.1097/CORR.0000000000002513 PMC10263201 36480637

[24] Global Burden of Disease Study 2013 Collaborators. Global, regional, and national incidence, prevalence, and years lived with disability for 301 acute and chronic diseases and injuries in 188 countries, 1990-2013: a systematic analysis for the Global Burden of Disease Study 2013. Lancet. 2015;386(9995):743–800. 26063472 10.1016/S0140-6736(15)60692-4 PMC4561509

[25] Centers for Disease Control and Prevention. National Ambulatory Medical Care Survey: 2010 Summary Tables 2010. Available from: https://www.cdc.gov/nchs/data/ahcd/namcs_summary/2010_namcs_web_tables.pdf. Accessed 16 May 2022.

[26] Demyttenaere K, Bruffaerts R, Lee S, Posada-Villa J, Kovess V, Angermeyer MC, Mental disorders among persons with chronic back or neck pain: results from the World Mental Health Surveys. Pain. 2007;129(3):332–42. 17350169 10.1016/j.pain.2007.01.022

[27] Beleckas CM, Prather H, Guattery J, Wright M, Kelly M, Calfee RP. Anxiety in the orthopedic patient: using PROMIS to assess mental health. Qual Life Res. 2018. 10.1007/s11136-018-1867-7 PMC6222016 29740783

[28] Celia D, Yount S, Rothrock N, Gershon R, Cook K, Reeve B, The Patient-Reported Outcomes Measurement Information System (PROMIS): progress of an NIH Roadmap cooperative group during its first two years. Med Care. 2007;45(5 Suppl 1):S3–S11. 10.1097/01.mlr.0000258615.42478.55 PMC2829758 17443116

[29] PROMIS. Depression: A brief guide to the PROMIS Depression instruments 2019. Available from: http://www.healthmeasures.net/images/PROMIS/manuals/PROMIS_Depression_Scoring_Manual.pdf.. Accessed 28 February 2019.

[30] PROMIS. Anxiety: A brief guide to the PROMIS Anxiety instruments 2019. Available from: http://www.healthmeasures.net/images/PROMIS/manuals/PROMIS_Anxiety_Scoring_Manual.pdf. Accessed 1 March 2019.

[31] Harris PA, Taylor R, Minor BL, Elliott V, Fernandez M, O’Neal L, The REDCap consortium: Building an international community of software platform partners. J Biomed Inform. 2019;95:103208. 31078660 10.1016/j.jbi.2019.103208 PMC7254481

[32] Harris PA, Taylor R, Thielke R, Payne J, Gonzalez N, Conde JG. Research electronic data capture (REDCap)–a metadata-driven methodology and workflow process for providing translational research informatics support. J Biomed Inform. 2009;42(2):377–81. 18929686 10.1016/j.jbi.2008.08.010 PMC2700030

[33] Baldry S. Wysa Receives FDA Breakthrough Device Designation for AI-led Mental Health Conversational Agent: Business Wire; 2022 [Available from: https://www.businesswire.com/news/home/20220512005084/en/Wysa-Receives-FDA-Breakthrough-Device-Designation-for-AI-led-Mental-Health-Conversational-Agent.

[34] Brooke J. SUS - A quick and dirty usability scale. Usability Eval Ind. 1996;189(194):4–7.

[35] Usability.gov. System Usability Scale (SUS): U.S. General Services Administration; 2022 [Available from: https://www.usability.gov/how-to-and-tools/methods/system-usability-scale.html.

[36] Firth J, Torous J, Nicholas J, Carney R, Rosenbaum S, Sarris J. Can smartphone mental health interventions reduce symptoms of anxiety? A meta-analysis of randomized controlled trials. J Affect Disord. 2017;218:15–22. 28456072 10.1016/j.jad.2017.04.046

[37] Karyotaki E, Efthimiou O, Miguel C, Bermpohl FMG, Furukawa TA, Cuijpers R Internet-Based Cognitive Behavioral Therapy for Depression: A Systematic Review and Individual Patient Data Network Meta-analysis. JAMA psychiatry. 2021 ;78(4):361–71. 33471111 10.1001/jamapsychiatry.2020.4364 PMC8027916

[38] Cuijpers P, Donker T, Johansson R, Mohr DC, van Straten A, Andersson G. Self-guided psychological treatment for depressive symptoms: a meta-analysis. PLoS One. 2011;6(6):e21274. 21712998 10.1371/journal.pone.0021274 PMC3119687

[39] Linardon J, Cuijpers P, Carlbring P, Messer M, Fuller-Tyszkiewicz M. The efficacy of app-supported smartphone interventions for mental health problems: a meta-analysis of randomized controlled trials. World psychiatry : official journal of the World Psychiatric Association (WPA). 2019;18(3):325–36. 31496095 10.1002/wps.20673 PMC6732686

[40] Carlbring P, Andersson G, Cuijpers P, Riper H, Hedman-Lagerlöf E. Internet-based vs. face-to-face cognitive behavior therapy for psychiatric and somatic disorders: an updated systematic review and meta-analysis. Cogn Behav Ther. 2018;47(1):1–18. 29215315 10.1080/16506073.2017.1401115

[41] Armontrout J, Torous J, Fisher M, Drogin E, Gutheil T. Mobile Mental Health: Navigating New Rules and Regulations for Digital Tools. Current psychiatry reports. 2016;18(10):91. 27553979 10.1007/s11920-016-0726-x

[42] Prodan A, Deimel L, Ahlqvist J, Birov S, Thiel R, Toivanen M, Success Factors for Scaling Up the Adoption of Digital Therapeutics Towards the Realization of P5 Medicine. Front Med (Lausanne). 2022;9:854665. 35492346 10.3389/fmed.2022.854665 PMC9039393

[43] Lord SE, Campbell ANC, Brunette MF, Cubillos L, Bartels SM, Torrey WC, Workshop on Implementation Science and Digital Therapeutics for Behavioral Health. JMIR mental health. 2021 ;8(1):e17662. 33507151 10.2196/17662 PMC7878106

[44] Carl JR, Jones DJ, Lindhiem OJ, Doss BD, Weingardt KR, Timmons AC, Regulating digital therapeutics for mental health: Opportunities, challenges, and the essential role of psychologists. Br J Clin Psychol. 2022;61 Suppl 1:130–5. 33650131 10.1111/bjc.12286

[45] Lese A, Sraj S. Rural Orthopedics: Providing Orthopedic Care in Rural Communities. Orthopedics. 2019;42(4):e350–e5. 31323106 10.3928/01477447-20190624-01

[46] Seifert A, Reinwand DA, Schlomann A. Designing and Using Digital Mental Health Interventions for Older Adults: Being Aware of Digital Inequality. Frontiers in psychiatry. 2019;10:568. 31447716 10.3389/fpsyt.2019.00568 PMC6696744

[47] Kenter RM, Cuijpers R Beekman A, van Straten A. Effectiveness of a Web-Based Guided Self-help Intervention for Outpatients With a Depressive Disorder: Short-term Results From a Randomized Controlled Trial. J Med Internet Res. 2016;18(3):e80. 27032449 10.2196/jmir.4861 PMC4832120

[48] Tung EL, Abramsohn EM, Boyd K, Makelarski JA, Beiser DG, Chou C, Impact of a Low-Intensity Resource Referral Intervention on Patients’ Knowledge, Beliefs, and Use of Community Resources: Results from the CommunityRx Trial. J Gen Intern Med. 2020;35(3):815–23. 10.1007/s11606-019-05530-5 PMC7080911 31749028

[49] De Jesús-Romero R, Wasil A, Lorenzo-Luaces L. Willingness to Use Internet-Based Versus Bibliotherapy Interventions in a Representative US Sample: Cross-sectional Survey Study. JMIR formative research. 2022;6(8):e39508. 36001373 10.2196/39508 PMC9453577

[50] Mongelli F, Georgakopoulos P, Pato MT. Challenges and Opportunities to Meet the Mental Health Needs of Underserved and Disenfranchised Populations in the United States. Focus (Am Psychiatr Publ). 2020;18(1):16–24. 32047393 10.1176/appi.focus.20190028 PMC7011222

[51] Thomas KC, Ellis AR, Konrad TR, Holzer CE, Morrissey JP. County-level estimates of mental health professional shortage in the United States. Psychiatr Serv. 2009;60(10):1323–8. 19797371 10.1176/ps.2009.60.10.1323

[52] Weil TP. Insufficient dollars and qualified personnel to meet United States mental health needs. The Journal of nervous and mental disease. 2015;203(4):233–40. 25816044 10.1097/NMD.0000000000000271

